# Fast polarization mechanisms in the uniaxial tungsten-bronze relaxor strontium barium niobate SBN-81

**DOI:** 10.1038/s41598-017-18252-7

**Published:** 2017-12-21

**Authors:** E. Buixaderas, C. Kadlec, M. Kempa, V. Bovtun, M. Savinov, P. Bednyakov, J. Hlinka, J. Dec

**Affiliations:** 1 Institute of Physics, Academy of Sciences of the Czech Republic, Na Slovance 2, 18221 Prague, Czech Republic; 2 Institute of Materials Science, University of Silesia, Pl-40-007 Katowice, Poland

## Abstract

The high-frequency dielectric response of the uniaxial strontium barium niobate crystals with 81% of Sr has been studied from 1 kHz to 30 THz along the polar *c* axis by means of several techniques (far infrared, time domain terahertz, high-frequency and low-frequency dielectric spectroscopies) in a wide temperature interval 20–600 K. Relaxor properties were observed in the complex dielectric response and four main excitations were ascertained below the phonon frequencies. These fast polarization mechanisms take place at THz, GHz and MHz ranges and show different temperature evolution. The central mode excitation in the THz range, related to anharmonic dynamics of cations, slightly softens from high temperatures and then hardens below *T* ~ 400 K. Below the phase transition (at *T* ~ 330 K) an additional microwave excitation appears near 10 GHz related to micro domain wall oscillations. The strongest relaxation appears in the GHz range and slows down on cooling according to the Arrhenius law. Finally, another relaxation, present in the MHz range at high temperatures, also slows down on cooling at least to the kHz range. These two relaxations are due to polar fluctuations and nanodomains dynamics. Altogether, the four excitations explain the dielectric permittivity maximum in the kHz range.

## Introduction

The relaxor compositions of the solid solution strontium barium niobate Sr_x_Ba_1−x_Nb_2_O_6_ (SBN) display compelling dielectric, pyroelectric, electro-optic and non-linear optical properties^1,2^. But, in addition, they present a great challenge in the understanding of the relaxor properties, in comparison to the better studied cubic perovskite relaxors^3^. SBN crystallizes in the unfilled tetragonal tungsten-bronze (TTB) structure in the compositional range 0.32 < *x < *82^4^. The TTB structure is a network of interconnected NbO_6_ octahedra forming three types of channels along the polar c axis which have different site symmetries: the A_1_ site (inside the squared channels) is occupied by Sr, the A_2_ site (inside the pentagonal ones) by Sr and Ba, and the C site (inside the triangular channels) is empty^5,6^. The intrinsic disorder present in the structure is the key to its excellent piezoelectric and dielectric properties^1,7^. The structure is highly anisotropic, as well as all its properties, and compositions with more than ~ 50% Sr show already mixed ferroelectric-relaxor properties. The composition with ~80% of Sr, SBN-80, displays a pronounced relaxor behaviour^8^: the dielectric permittivity displays a broad maximum in *ε*′(*T*) at the temperature *T*_m_ ~ 300 K in the sub-hertz range which shifts up to 350 K at several megahertzs, and presumably to higher temperatures at higher frequencies. This strong dielectric dispersion, observed mainly at temperatures around the *ε*′(*T*) maximum, is the main characteristic of the relaxor behaviour^9,10^. At very high temperatures SBN-80 should show the centrosymmetric point group 4/*mmm* ^11^, as other TTBs, although its structure was not actually measured at such high temperatures. Nevertheless, modulations in the position of oxygen atoms are present up to at least 700 K^12^. On cooling and below *T*_m_, a tetragonal phase appears, with the space group *P*4*bm* ^6^ and spontaneous polarization along the *c* axis, as in other members of the family with lower Sr content. However, depending on the crystallization method an orthorhombic distortion could appear in some crystals with high Sr content^5,13^. The size of the ferroelectric domains is dependent on the Sr content and, for Sr_0.75_Ba_0.25_Nb_2_O_6_ (SBN-75), the domain size is of the order of 100 nm^14^. Recently it was found that polar nanoregions in SBN-80 show a dynamics in the low-frequency range, namely their transverse and longitudinal breathing, and their transformation into polar nanodomains (PNDs)^8^. However, no dielectric data were published at higher frequencies, and therefore faster polarization mechanisms were not included in the picture of the dielectric response of high-Sr content SBN. We already reported the far infrared (IR) reflectivity spectrum of Sr_0.61_Ba_0.39_Nb_2_O_6_ (SBN-61) and the temperature dependence of its IR active modes along the polar axis^15^, as well as its broadband dielectric behaviour (from 100 Hz to 67 THz). In this composition, the slowing down of a strong relaxation, together with a THz central mode, is responsible for the huge dielectric anomaly below phonon frequencies.

The fast dynamics of the phase transition in the SBN structure was studied by Raman spectroscopy for crystals with several compositions^16–19^. A proper soft mode was never found; therefore, an order-disorder mechanism of the transition was implied. In agreement with this, central peaks at very high temperatures were discovered by Brillouin^20^ and neutron scattering^21^, indicating the thermal switching of PNDs and the anharmonic hopping motion of Ba/Sr atoms. Inelastic neutron scattering on SBN-61 confirmed the relaxation of polar fluctuations in the *ab* plane, with a correlation length of 5–10 unit cells, responsible for the dielectric anomaly^22,23^. These data show that a complex interaction between slow and fast polarization mechanisms is responsible for the phase transition in TTBs.

The dielectric behaviour of the relaxor compositions of SBN should be very dependent on the formation of polar regions of different sizes seen by light scattering^24^ and transmission electron microscopy^25^, but up to now no comprehensive and exhaustive dielectric studies were performed in the relaxor compositions. Therefore, in this paper, we report on the dielectric response from 10^3^ to 10^13^ Hz along the polar *c* axis of the utmost uniaxial SBN relaxor with 81% of Sr (SBN-81), in the temperature range 20 to 600 K, using different spectroscopic techniques. This broadband approach is able to unveil the main excitations responsible for the fast mechanisms of the polarization, and elucidate their relationship to the relaxor behaviour.

## Experimental Results

### IR and THz measurements

**Figure 1:**
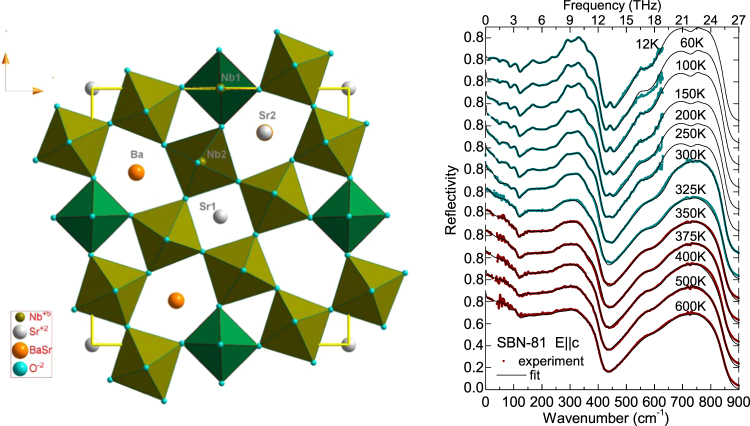
Structure of SBN in the paraelectric phase in the *ab* plane and IR reflectivity spectra of SBN-81 (dotted) together with their fits (lines) at several temperatures for the E||c polarization, including the experimental TDTTS data in the THz range. Red spectra are taken in the paraelectric phase, blue in the ferroelectric one.

The unit cell of SBN, presented in Fig. 1, has five (Sr_*x*_Ba_1−*x*_)Nb_2_O_6_ units, and contains 45 atoms, resulting in the formula (Sr_*x*_Ba_1−*x*_)_5_Nb_10_O_30_, due the vacancies present in the channels. In the paraelectric phase, the theoretical analysis for the centrosymmetric space group *P*4/*mmm* predicts 138 phonon modes at the centre of the Brillouin zone^11,15^:

$$\begin{array}{c}{{\rm{\Gamma }}}^{{\rm{T}}{\rm{E}}{\rm{T}}}(4/mmm):9\,{{\rm{A}}}_{2{\rm{u}}}({\rm{c}};-)+10\,{{\rm{A}}}_{2{\rm{g}}}(-;-)+5\,{{\rm{A}}}_{1{\rm{u}}}(-;-)\\ \quad \quad \quad \quad \quad \quad +10\,{{\rm{A}}}_{1{\rm{g}}}(-;{a}^{2}+{b}^{2},{c}^{2})+8{{\rm{B}}}_{1{\rm{u}}}(-;-)\\ \quad \quad \quad \quad \quad \quad +10\,{{\rm{B}}}_{1{\rm{g}}}(-;{a}^{2}-{b}^{2})+4\,{{\rm{B}}}_{2{\rm{u}}}(-;-)\\ \quad \quad \quad \quad \quad \quad +10\,{{\rm{B}}}_{2{\rm{g}}}(-;ab)+26\,{{\rm{E}}}_{{\rm{u}}}({\rm{a}},{\rm{b}};-)+10\,{{\rm{E}}}_{{\rm{g}}}(-;ac\,,bc).\end{array}$$

Out of them, 1A_2u_ + 1E_u_ are acoustic modes, and E_u_ and E_g_ modes are double degenerated. IR and Raman activities are shown in parentheses. The site group analysis for the ferroelectric phase, using Jamieson data for SBN-75 at room temperature – point group 4 *mm* ^5^, with an effective Ba/Sr atom in the pentagonal site 4*c* and without disorder among O atoms, gives the following active modes^5^:

$$\begin{array}{c}{{\rm{\Gamma }}}^{{\rm{T}}{\rm{E}}{\rm{T}}}{(4mm)}_{{\rm{o}}{\rm{r}}{\rm{d}}{\rm{e}}{\rm{r}}{\rm{e}}{\rm{d}}}:19\,{{\rm{A}}}_{1}({\rm{c}};{a}^{2}+{b}^{2},{c}^{2})+14\,{{\rm{B}}}_{1}(-;{a}^{2}-{b}^{2})\\ \quad \quad \quad \quad \quad \quad \quad \,\,+15{{\rm{A}}}_{2}(-;-)+18\,{{\rm{B}}}_{2}(-;ab)+36\,{\rm{E}}({\rm{a}},\,{\rm{b}};ac,bc)\end{array}$$

All modes are optical, except 1 A_1_ + 1 E, which are acoustic, E modes being double degenerated. However, taking into account oxygen disorder, we obtain 168 active modes distributed into the following phonon symmetries^15^:

$$\begin{array}{c}{{\rm{\Gamma }}}^{{\rm{T}}{\rm{E}}{\rm{T}}}{(4mm)}_{{\rm{d}}{\rm{i}}{\rm{s}}{\rm{o}}{\rm{r}}{\rm{d}}{\rm{e}}{\rm{r}}{\rm{e}}{\rm{d}}}:23\,{{\rm{A}}}_{1}({\rm{c}};{a}^{2}+{b}^{2},{c}^{2})+18\,{{\rm{B}}}_{1}(-;{a}^{2}-{b}^{2})+19\,{{\rm{A}}}_{2}(-;-)\\ \quad \quad \quad \quad \quad \quad \quad \,\,\,\,\,\,+22\,{B}_{2}(-;ab)+43\,{\rm{E}}({\rm{a}}\,,{\rm{b}};ac,bc).\end{array}$$

External phonon modes due to Ba and Sr vibrations are present in the IR and Raman spectra below ν ~ 180 cm^−1^. At higher wavenumbers, phonons are due to Nb-O stretching and O-Nb-O bending vibrations in the octahedral network^26^. In the case that SBN had an orthorhombic distortion, the expected point group is *mm*2 and the 138 phonon modes would be distributed in the following symmetries:

$${{\rm{\Gamma }}}^{{\rm{ORT}}}{(mm2)}_{{\rm{ordered}}}:33\,{{\rm{A}}}_{1}({\rm{c}};{a}^{2},{b}^{2},{c}^{2})+33\,{{\rm{A}}}_{2}(;\,ab)+36\,{{\rm{B}}}_{1}({\rm{a}};ac)+36{{\rm{B}}}_{2}({\rm{b}};bc).$$

Out of them, 1A_1_ + 1B_1_ + 1B_2_ would be acoustic.

The IR reflectivity spectra of vibrations of SBN-81 along the polar axis, **E**||**c**, corresponding to modes of A_2u_/A_1_ symmetry, are depicted in the right panel of Fig. 1. Spectra –including the THz reflectivity points calculated from the time-domain THz transmission spectroscopy (TDTTS) experiment– are shown together with the corresponding fits at selected temperatures. The experimental reflectivity spectra were fitted with the generalized oscillator model of the dielectric function^27^,

1a $${\hat{\varepsilon }}_{c}(\nu )=\varepsilon ^{\prime} (\nu )-{\rm{i}}\varepsilon ^{\prime\prime} (\nu )={\varepsilon }_{\infty }\prod _{i=1}^{n}\frac{{\nu }_{LOi}^{2}-{\nu }^{2}+{\rm{i}}\nu {\gamma }_{LOi}}{{\nu }_{TOi}^{2}-{\nu }^{2}+{\rm{i}}\nu {\gamma }_{TOi}},$$

1b $${\rm{\Delta }}{\varepsilon }_{i}=\frac{{\varepsilon }_{\infty }}{{\nu }_{TOi}^{2}}\frac{\prod _{k}({\nu }_{LOk}^{2}-{\nu }_{TOi}^{2})}{\prod _{k\ne i}({\nu }_{TOk}^{2}-{\nu }_{TOi}^{2})},$$

where ε_∞_ is the permittivity at frequencies much higher than all polar phonon frequencies, ν_*TOi*_ and ν_*LOi*_ are the transverse and longitudinal frequencies of the *i*-th phonon mode and γ_*TOi*_ and γ_*LOi*_ their respective damping constants. The dielectric strength of each mode Δε_*i*_ is then automatically calculated by eq. (1b).

In the paraelectric phase, 11 A_2u_ modes were fitted (3 external modes from cations plus 7 internal ones and one overdamped low-frequency mode in the THz range). Theoretically 8 A_2u_(c) optical modes are allowed, after subtracting the acoustic branch, but the double occupancy of some sites and disorder in oxygen positions enable the activation of more modes. In the ferroelectric phase, 20 A_1_ modes were fitted at the lowest temperature (5 external modes, 14 internal modes and a low-frequency one in the THz range); although 22 A_1_ are predicted for the tetragonal phase, and 33 in the case of the orthorhombic variation. According to the reflectivity results, our SBN-81 crystal is macroscopically tetragonal, as there is no evident splitting of phonons even at the lowest temperatures, like in orthorhombic Ba_2_NaNb_5_O_15_^28^. Internal modes are overlapped inside the highest frequency band; therefore, the parameters of these high-frequency modes are rather estimations.

Phonon frequencies above ν = 50 cm^−1^ show no significant anomalies on cooling. Below *T*_m_ several phonons appear, favouring the order-disorder picture of the ferroelectric phase transition, as in SBN-61^15^. The overdamped THz central mode shows a pronounced softening towards *T*_m_, therefore, it is partially responsible for the phase transition.

Table 1 presents the list of the fitted phonon parameters –transverse frequency ν_T_, transverse damping γ_T_, and dielectric strength Δε−, calculated from (1b), in the paraelectric (400 K) and in the ferroelectric (12 K) phases. Δε of the lowest-frequency mode, in the THz range, changes from 570 at 400 K to 12 at 12 K, and its frequency rises from 20 to 47 cm^−1^. In the ferroelectric phase new modes, stemming from the formerly Raman active modes in the paraelectric phase, appear. The number of modes and the spectral shape are in agreement with the tetragonal symmetry, as in SBN-61.

**Table 1:** Parameters of the IR phonons of SBN-81 for the E||c polarization in the paraelectric and ferroelectric phases (ν_T_ and γ _T_ in cm^−1^).

	SBN-81 *E*||*c A*_1_/*A*_2u_ modes					
	Ferroelectric phase (12 K)			Paraelectric phase (400 K)		
	ν_T_	γ_T_	Δε	ν_T_	γ_T_	Δε
ν_THz_	46.9	32.0	12	19.8	52.4	570
External modes	61.3	26.7	14.0	75.0	66.5	2.7
	80.3	20.9	4.0			
	109.7	37.0	10.0	108.8	43.2	3.3
	133.4	48.6	7.0	148.6	78.9	10.7
	166.8	28.9	0.1			
Internal modes	204.6	32.3	0.6	193.7	64.2	1.7
	237.0	39.1	3.8			
	264.5	30.1	2.6	256.0	80.9	5.0
	284.5	21.1	1.0	291.0	100.0	3.5
	309.2	26.3	0.02			
	327.7	27.4	0.5			
	351.9	24.0	0.6	360.5	97.0	0.6
	358.5	65.0	1.0			
	444.0	20.4	0.1			
	542.1	43.8	0.7	557.0	91.2	2.0
	622.1	58.3	2.7	637.0	95.0	1.4
	736.0	44.1	0.03	735.0	52.0	0.01
	775.0	46.0	0.01			
	904.0	63.0	0.02			

**Figure 2:**
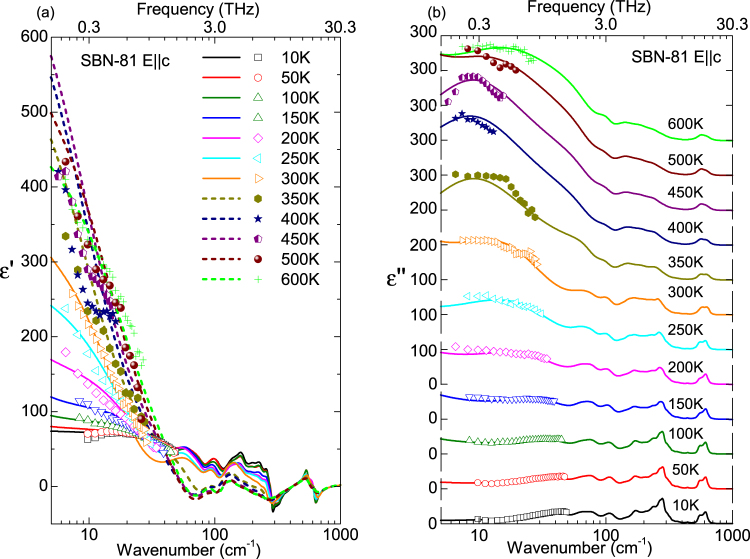
Permittivity (**a**) and dielectric loss (**b**) functions of SBN-81 obtained from the far IR fit of the reflectivity at different temperatures, together with the points measured in the TDTTS experiment.

The permittivity and losses calculated from the previous fits are presented together with the experimental TDTTS points in Fig. 2, at selected temperatures. The permittivity along the polar axis due to the phonon contribution is rather low (Δε_ph_ ~ 40), as in other TTB materials^15,28^, but the THz central mode enhances dramatically the ε′ values near 10 cm^−1^ at high temperatures, as seen in Fig. 2a. On cooling, this excitation loses its dielectric strength and the THz values of the permittivity agree with the phonon contribution. In Fig. 2b the dielectric loss spectra are displayed at different temperatures. Each peak corresponds to a phonon mode. On cooling, phonons become better distinguished and the intense THz central mode, seen as a broad peak at high temperatures, gradually weakens and shifts to higher frequency. The oscillator model of eq. (1) used for this fitting, combining reflectivity, permittivity and dielectric loss in the THz range, lacks the influence of the lower frequency relaxations on the GHz range and below, therefore another fit was performed (see Discussion), taking into account lower frequency data, down to kHz. Nevertheless, the model of eq. (1) gives more reliable phonon parameters with reasonable values in the THz range.

### Low-and high-frequency dielectric measurements

**Figure 3:**
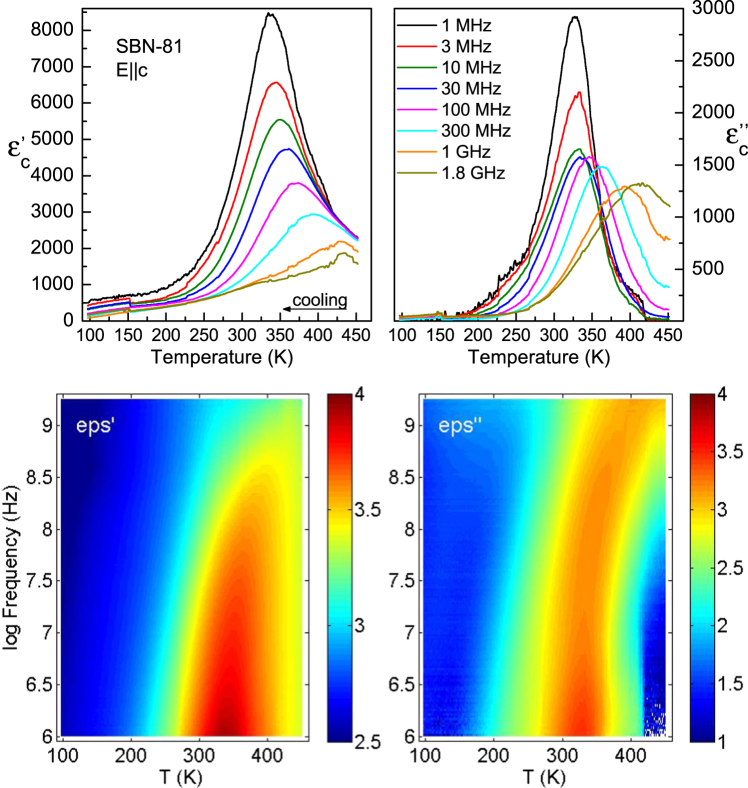
Upper panels: Temperature dependence of the permittivity (left) and dielectric loss (right) of SBN-81 measured at high frequencies on cooling. Lower panels: 2D intensity maps of the dielectric data –permittivity (left), dielectric loss (right) –in log scale versus temperature and frequency. Colors are illustrative, dark red meaning the highest value.

The complex dielectric function ${\hat{\varepsilon }}_{c}(T,\nu )=\varepsilon ^{\prime} -{\rm{i}}\varepsilon ^{\prime\prime}$ at high-frequencies obtained by the coaxial technique on cooling is shown in Fig. 3. Upper panels show the strong dispersion of the permittivity and dielectric loss maxima at *T*_m_, shifting from 400 K at 1.8 GHz to 330 K at 1 MHz, and it reveals the strong relaxor behaviour of the crystal along the polar axis. The complete set of measured data, with all the frequencies is displayed in the lower panels in the form of 2D intensity maps, where a broad maximum crossing the GHz–MHz ranges is well visible with maximum of intensity around 330 K at megahertzs. The dielectric loss spectrum versus frequency is shown in the lower right panel, where a slowing down of the main relaxation is revealed.

**Figure 4:**
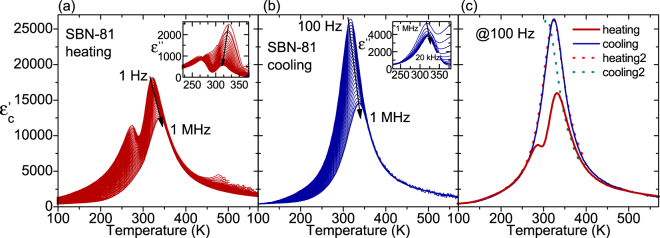
Dielectric measurements at low frequencies of SBN-81 on heating and cooling.

The dielectric measurements up to 10^6^ Hz show that there is an important pre-history effect in the dielectric response of the SBN-81 crystal (see Fig. 4). On heating, apart from the main dielectric anomaly near 330 K, we found other anomalies, visible below 1 kHz, at about 280 and 480 K (Fig. 4a). When heating the sample to 600 K and then cooling again (Fig. 4b), these extra anomalies are not seen, just the main anomaly at *T*_m_ is present. It seems that the heating erases the thermal and cluster pre-history and transforms the sample into a virgin-like state. After the heating up to 600 K and during the subsequent cooling, the sample showed much higher conductivity. Figure 4c shows the thermal hysteresis of the permittivity at 100 Hz for two consecutive heating-cooling runs. Due to these effects on the values of ε′, the interpretation of the data at very low frequencies are left out of the scope of this paper.

**Figure 5:**
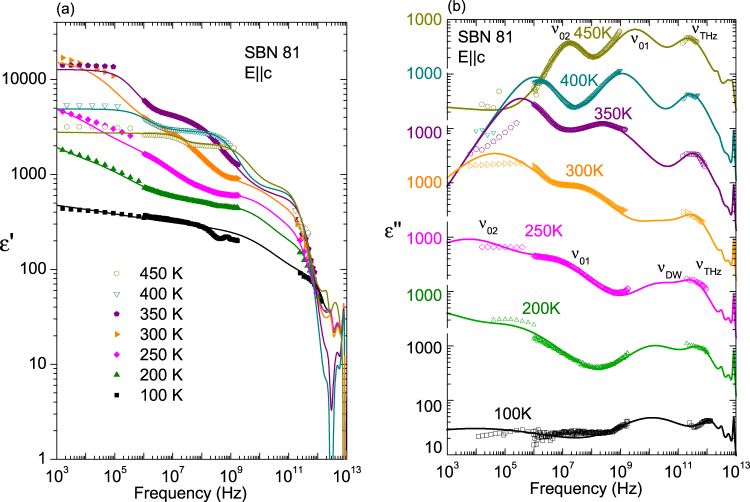
Selected frequency cuts of the permittivity and dielectric losses of SBN-81 from the dielectric and coaxial line experiments, together with the experimental points from the TDTTS experiment, and fits of the data using several relaxations. Note the logarithmic scales.

To better understand the frequency dependence of the complex permittivity, we plot together dielectric spectra obtained by the dielectric and TDTTS experiments for selected temperatures. The broad-band experimental dielectric spectra are presented in Fig. 5 with the fits described in the Discussion section. On cooling from 470 K, the low-frequency permittivity rises towards *T*_m_ and then decreases monotonically. Maxima of the dielectric loss spectra allow us to identify the main excitations in the crystal. Three distinct peaks are seen at high temperatures in the paraelectric phase. The peak located at the THz range –labelled ν_THz_– corresponds to the central mode measured in the THz-far IR spectra. The maxima of the other two relaxations –labelled ν_01_ and ν_02_, respectively– shift to lower frequencies on cooling. The lowest frequency one, ν_02_, shifts below the MHz range at about 350 K. Due to the limited amount of data from the dielectric loss spectra at these frequencies, ν_02_ is difficult to assess and its parameters are taken only as estimations. On further cooling, below *T*_m_, a new excitation (ν_DW_) appears near 10 GHz. This excitation is needed to reach the permittivity values at lower frequencies and to fit the increase of the dielectric loss data signalized by the slope near the GHz range in Fig. 5b.

## Discussion

To evaluate the behaviour of all the excitations on cooling, we fit the dielectric spectra below phonons at several temperatures with a model consisting of a sum of several Cole-Cole terms for the excitations below THz, a damped oscillator for the central mode in the THz range, and the phonon contribution:

2 $$\hat{\varepsilon }(\nu )=\varepsilon {\rm{^{\prime} }}(\nu )-{\rm{i}}\varepsilon {\rm{^{\prime} }}{\rm{^{\prime} }}(\nu )=\sum _{j}\frac{{\rm{\Delta }}{\varepsilon }_{0j}}{1+{({\rm{i}}\nu /{\nu }_{0j})}^{1-{\alpha }_{j}}}+\frac{{\rm{\Delta }}{\varepsilon }_{THz}{\nu }_{TH{\rm{z}}}^{2}}{{\nu }_{TH{\rm{z}}}^{2}-{\nu }^{2}+{\rm{i}}{\gamma }_{TH{\rm{z}}}\nu }+{\rm{\Delta }}{\varepsilon }_{ph}$$

here, Δε_0*j*_ denotes the dielectric strength of the *j*-th excitation, ν_0*j*_ its frequency, *α*_*j*_ is a real index between 0 and 1, which determines the deviation from the pure Debye model for the relaxations and gives a notion of the frequency width of the loss peak. In the oscillator term, γ_*THz*_ refers to its damping constant, and Δε_*ph*_ stands for the overall contribution of harmonic phonons at higher frequencies, which is about 40.

**Figure 6:**
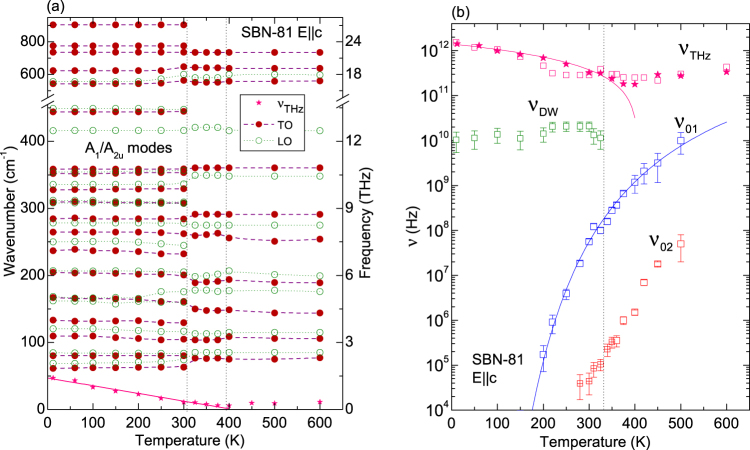
(**a**) Temperature dependence of the IR active phonon frequencies (transverse–TO and longitudinal–LO) in SBN-81 along the polar axis, and (**b**) temperature dependence of the main relaxations found below phonons. The excitation in the THz range is presented in both panels (stars – from the fit (1), squares - from the fit using (2). The relaxation ν_02_ refers to that found in ref.^8^ near *T*_m_ and labelled there *f*_c_ (crossed squares). Lines correspond to the fits: (3) for ν_THz_ and (4) for ν_01_.

In Fig. 6a we show the temperature dependence of the polar phonon frequencies detected by the far IR reflectivity experiment and corresponding to the fits using eq. (1). The mean relaxation frequencies found by TDTTS and the dielectric spectroscopies, corresponding to the fits with eq. (2), are shown in Fig. 6b. The phase transition is well detected by phonons between 300 and 325 K. New phonons are activated in the non-centrosymmetric phase. In the THz range, below 50 cm^−1^, there is an overdamped mode which shows an anomaly near 400 K. This mode was fitted with the renormalized law

3 $${\nu }_{{\rm{r}}}(T)=a({T}_{{\rm{m}}}-T)$$

below 400 K; this dependence does not correspond to the classic soft mode behaviour, which evidences that this vibration is not related to the harmonic vibration of an atom, but rather to the anharmonic hopping of disordered cations (Sr, Ba)^21^. The fitting parameters of this excitation are *T*_m_ ~ 409 K and *a = *3.4 × 10^9^ s^−1^K^−1^. Above *T*_m_ there are not enough points to perform a reliable fit, but from Fig. 2b it is evident that the frequency of this excitation hardens on heating from *T*_m_. In analogy to SBN-61 and other relaxor ferroelectrics, it can be expected that, on heating, ν_THz_ increases, merges with phonons in the form of a central mode and, finally, disappears above the Burns temperature, where the PNDs vanish (above 580 K in the case of SBN)^9,15^.

In Fig. 6b we show the excitations present below phonons. Apart from the THz central mode, there are several excitations at lower frequencies. Because of experimental limitations, we can follow them properly only below 480 K, although they should be present also above this temperature. At high temperatures, in the paraelectric phase, there are probably two excitations: the THz central mode ν_THz_ and the MHz-GHz range relaxation ν_01,_ which contributes to the permittivity values measured in the dielectric experiment, and is related to dynamics of the polar fluctuations^9^. On cooling both excitations should slow down and, below 500 K, a third excitation, ν_02,_ is also detected. This relaxation slows down to 100 kHz range at 350 K and corresponds to the relaxation *f*_c_ found in^8^, assigned to the headways breathing of PNDs along the polar axis. We see it at higher temperatures and frequencies, but on cooling it goes out from our frequency window slowing down further into the kHz range. On further cooling, below about 330 K, the fourth excitation ν_DW_ appears near 10 GHz_._

The relaxations ν_01_ and ν_02_ slow down from the paraelectric phase, but the excitations ν_THz_ and ν_DW_ display different temperature dependences. ν_THz_ softens towards ~400 K and then hardens, as described above; while ν_DW_ appears below *T*_m_ and exhibits only a slight temperature dependence. ν_DW_ correlates with the presence of diffuse scattering, as found in SBN-70^24^ and in SBN-61^23^. As it is needed to explain the permittivity values only below *T*_m_, it is probably due to oscillations of the tiny ferroelectric domains appearing in the crystal, as demonstrated by the partial vanishing of the diffuse scattering by poling^23,24^ and by recent studies of the dielectric response of other disordered ferroelectrics^29^.

**Figure 7:**
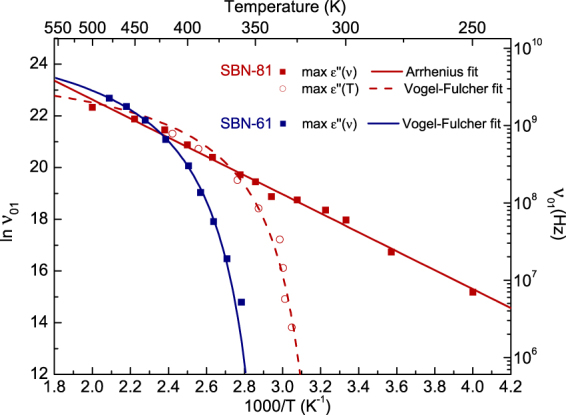
Fits of the maxima of the dielectric loss for the relaxation ν_01_ in the GHz range. Maxima of ε″(ν)–red squares, maxima of ε″(*T*)–red circles for SBN-81. Maxima of ε″(ν)–blue squares for SBN-61. Lines correspond to fits with eqs (4) or (5).

Usually, the relaxor behaviour of a material is verified by the analysis of the temperature dependences of the mean relaxation frequency and checking whether it follows the classic Arrhenius law

4 $$\nu (T)={\nu }_{{\rm{\infty }}}\exp (-{E}_{a}/\kappa T),$$

or the Vogel-Fulcher law, with a freezing temperature *T*_VF_:

5 $$\nu (T)={\nu }_{{\rm{\infty }}}\exp (-{E}_{a}/\kappa (T-{T}_{{\rm{V}}{\rm{F}}})),$$

where ν_∞_ is the saturation frequency, *E*_*a*_ the activation energy and κ the Boltzmann constant κ = 8.617·10^−5^ eVK^−1^. Figure 7 shows the ν(*T*) dependence for the main relaxation, ν_01_, obtained from the positions of the dielectric loss maxima in both ε″(ν) and ε″(*T*) shown in Figs 3 and 5b respectively. Although the ε″(ν) and ε″(*T*) dependences are obtained from the same set of experimental data, the ν(*T*) shape is dependent on the selection of ε″(ν) or ε″(*T*) maxima, and it could be misleading. In our case, the ν(*T*) plot based on the ε″(ν) maxima follows the Arrhenius law, while the plot based on the ε″(*T*) maxima follows the Vogel-Fulcher law. The Arrhenius fit parameters are ν_∞_ = 10 ± 3 THz and E_*a*_/κ = 3.7 ± 0.1 K, whereas the Vogel-Fulcher fit parameters of ε″(*T*) are ν_∞_ = (20 ± 5) GHz, E_*a*_/κ = 236 ± 30 K and *T*_VF_ = 303 ± 4 K. Such a discrepancy is usual for relaxor ferroelectrics (see^30^, for instance) and reflects the fact that the dielectric strength of the main relaxation in relaxors is decreasing on cooling. Therefore, the correct way to estimate ν(*T*) is using the ε″(ν) spectra measured at a constant temperature. Consequently, the Arrhenius law for the ν_01_ relaxation is reliable: the PNDs responsible for the relaxation ν_01_ in SBN-81 actually do not freeze and the activation energy of their fluctuations is much lower than in SBN-61. For comparison, the ε″(ν) maxima in SBN-61^15^ are also displayed in Fig. 7. The fit with eq. (5) [ν_∞_ = (14.0 ± 0.5) GHz, E_*a*_/κ = 270 ± 4 K and *T*_VF_ = 330 K] shows the qualitative difference between both crystals: ν(*T*) follows the Arrhenius law in SBN-81 but a proper Vogel-Fulcher law in SBN-61. Dielectric measurements in SBN-75^31^ found that the main relaxation responsible for the phase transition at *T*_m_ ~ 310 K has also an Arrhenius behaviour with shorter relaxation time τ = 5.4·10^−16^ s (ν = 1.9·10^15^ Hz), but there is another relaxation at lower temperatures with τ = 1.8·10^−11^ s (ν = 5.6·10^10^ Hz). This is in agreement with the central peak and the relaxation time found in Brillouin scattering^32^, as well as with the oscillation of tiny domain walls (of size smaller than 1 nm) found in the quasielastic neutron scattering of SBN-70^24^, and also with the excitation ν_DW_ we found near 10 GHz.

**Figure 8:**
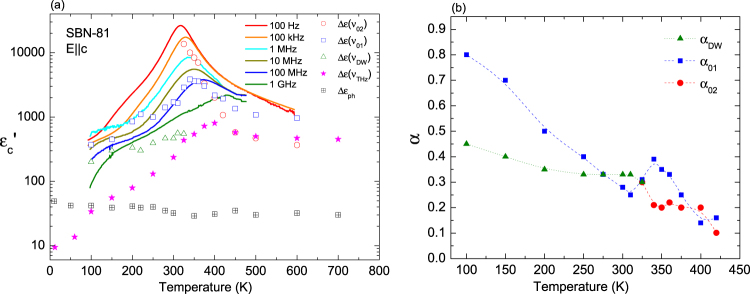
(**a**) Permittivity measured at several frequencies and dielectric contribution of the different excitations found in SBN-81 along the polar axis. (**b**) α parameters of the Cole-Cole fit used for the three main excitations below the central mode ν_THz_.

The temperature dependence of the permittivity of the SBN-81 crystal along the polar axis is displayed in Fig. 8a for various frequencies from 100 Hz to GHz, together with the dielectric contributions of the main excitations from the fit with eq. (2). The maximum of permittivity related to *T*_m_ shifts from THz to kHz, exhibiting the relaxor character. THz data show a quite marked temperature dependence, but the contribution of the overall phonons is small (Δε_ph_ ~ 40) and relatively temperature independent. The behaviour of the dielectric strength of ν_01_ demonstrates that ν_01_ is responsible for the dielectric anomaly at *T*_m_ in the GHz–MHz range; however, its dielectric contribution is not enough to explain the permittivity at lower frequencies, where the relaxation ν_02_ emerges.

The α parameters of the three Cole-Cole relaxations are presented in Fig. 8b. This parameter estimates the deviation of a relaxation from the Debye spectrum and the width of the relaxation frequency distribution. The relaxation ν_01_ shows an important broadening on cooling from high temperatures and also a step in α near *T*_m_, meaning that its character is affected by the phase transition, although its frequency does not show a remarkable anomaly. Below *T*_m_, the excitation ν_DW_ is needed to fit the microwaves data, but, as we do not have precise results in the GHz range, its α parameter (α_DW_) is an estimation. Nevertheless, the value α_DW_ ~ 0.3–0.4, which slightly increases towards low temperatures, corresponds well to the attribution of the ν_DW_ excitation to domain walls oscillations. The α parameter of ν_02_ is relatively small at high temperatures (α_02_ ~ 0.1), showing near Debye character, but it increases on cooling.

These results indicate the presence of several polarization mechanisms contributing to the dielectric response of SBN-81 at high frequencies. Their interplay is responsible for the complicated relaxor-ferroelectric behaviour. Each mechanism should be associated with some microscopic object in the lattice crystal. As known from literature and experiments^6,21,22,24,25^, several phenomena coexist in SBN: hopping of disordered cations, incommensurations in the positions of oxygen atoms, polar fluctuations and PNDs. All of them contribute to the permittivity in different length scales and, consequently, in different frequency and temperature ranges. In comparison with SBN-61, SBN-81 has a variety of fast polarization mechanisms with different correlation lengths below the THz central mode, not only a strong relaxation with a huge slowing down^15^.

The low-frequency processes below 1 kHz are out of our analysis: dielectric measurements near 100 Hz already showed that the experimental data are dependent on the thermal prehistory of the crystal, the geometric shape of the sample, the measuring field, heating-cooling rates, etc.; therefore, we focused on higher frequency excitations to be able to compare these data with data taken on SBN-61. Our experimental results are complementary and in agreement with the dielectric experiments done at lower frequencies, where slow polarization mechanisms were discussed. Our relaxation ν_02_ is the relaxation *f*_*c*_ found in ref.^8^ just seen at higher temperatures and frequencies and the excitation ν_DW_ confirms the existence of the actual ferroelectric phase transition at ~330 K. Below ν_02_ (or *f*_*c*_) there is a slower relaxation *f*_*a*_, in the sub-Hertz range, which dominates in permittivity near the phase transition, and it was interpreted as the sideways breathing of the PNDs. These two relaxations (*f*_*c*_ and *f*_*a*_) display a dynamic scaling of the 3D Random-Field Ising model^8,33^ and evidence the occurrence of a phase transition from the relaxor state to the ferroelectric one with actual ferroelectric domains.

The evolution of the fast polarization dynamics in SBN-81 in the sub-phonon range can be summarized like this: as in other relaxors, a strong excitation in the THz range is assumed above the Burns temperature *T*_d_. On cooling it splits into three components: an anharmonic vibration in the THz range (the central mode ν_THz_, related to anharmonic hopping of cations^21^), a strong GHz relaxation (ν_01_) associated to polar fluctuations or flipping of the forming PNDs^9^ and a lower-frequency relaxation ν_02,_ related to breathing of PNDs^8^. On cooling, these relaxations become stronger, showing maxima of their contribution to the dielectric permittivity at different temperatures. The anharmonic hopping connected to ν_THz_ softens to ~400 K and then hardens, but the relaxations ν_01_ and ν_02_ slow down further, showing the maximum of the dielectric contribution gradually at ~350 K and 330 K, respectively. Below 330 K, in the ferroelectric phase, ν_DW_ appears in the GHz range and all the contributions eventually weaken. The coexistence of different polarization mechanisms gives rise to the overall dielectric dispersion, which is different from the huge and single mechanism present in SBN-61^15^. The onset of the phase transition occurs near 400 K and then, on cooling, several mechanisms with different correlation lengths contribute to the developing of ferroelectricity. The crystal reaches finally the ferroelectric state below 330 K.

## Conclusions

The high-frequency dielectric response of SBN-81 single crystal along the polar axis has been studied from 10^3^ to 10^13^ Hz by several experimental techniques in a broad-band approach. The phase transition was revealed by far infrared spectroscopy near 330 K. No classical soft phonon mode was found, although new phonons appear below *T*_m_, which favours the order-disorder mechanism of the phase transition. Notwithstanding, an anharmonic excitation in the THz range, a central mode, softens towards 400 K, following a linear law. Due to the presence of anharmonic disorder in the crystal, it was assigned to hopping of Sr/Ba cations inside the channels of the NbO_6_ network, as in SBN-61.

Several relaxations, corresponding to fast polarization mechanisms below the phonon frequencies, play an essential role in the dielectric response of the crystal. The main contribution to the permittivity comes from a strong relaxation (ν_01_) present in the GHz range at high temperatures which slows down on cooling following the Arrhenius law. A second relaxation with lower frequency (ν_02_) slows down, as well, contributing to the permittivity mainly near *T*_m_. Both these relaxations can be assigned to polar fluctuations, probably flipping (ν_01_) and breathing (ν_02_) of polar nanodomains. The excitation that appears below *T*_m_ around 10 GHz (ν_DW_) is associated to the development of ferroelectric microdomains. Altogether, the four mechanisms explain, above the kHz range, the ferroelectric transition in SBN-81 as well as its relaxor character, which differs from the behaviour displayed by SBN-61 and lead-based relaxors.

## Methods

The SBN-81 single crystal was grown by the Czochralski method, as in ref.^8^. The real composition of our crystal was determined using Inductively Coupled Plasma-Optical Emission Spectroscopy and corresponds to Sr_0.81_Ba_0.17_Nb_2_O_5.98_. The possible deviation from the nominal stoichiometry is within the experimental uncertainty and is not expected to have any significant influence on our high-frequency dielectric results or on the polarization mechanism discussed in the paper. A big bulk crystal was cut in several samples with different geometries and these were studied by means of Fourier-transform IR reflectivity, time-domain THz transmission spectroscopy (TDTTS) from 0.1 to 2.5 THz, high-frequency coaxial line technique (1 MHz–1.8 GHz), and low-frequency dielectric measurements (100 Hz–1 MHz).

IR reflectivity measurements were performed on a plate (4.5 × 5 × 0.8 mm^3^, with the polar axis oriented along one of the edges of the plate) using a Fourier spectrometer Bruker IFS 113v equipped with two room temperature DTGS pyroelectric detectors as well as He-cooled (1.5 K) Si bolometer. The light from a Hg lamp was polarized by a metal-mesh polarizer deposited on a thin polyethylene foil. For low temperature measurements (down to 12 K) a continuous-flow Oxford Optistat CF cryostat was used and the sample was mounted in a He gas bath, and for high temperatures (300–600 K) we used a custom-made oven. Room and high temperature spectra were measured in the range 30–1800 cm^−1^ (1–60 THz) with a resolution of 2 cm^−1^. Due to the presence of polarizers and optical windows the accessible frequency range at low temperatures was 30–620 cm^−1^.

TDTTS measurements were carried out on a thin polished plane-parallel sample (4.5 × 5 × 0.05 mm^3^) with orientation (100) in the temperature range 10–800 K, using a polarized electromagnetic field to measure the ***E***||***c*** spectra. A custom-made time-domain THz transmission spectrometer was used to obtain the complex dielectric response from 3 to 50 cm^−1^ with a resolution of 0.5 cm^−1^. An Optistat CF cryostat with Mylar windows was used for measurements down to 10 K. An adapted commercial high-temperature cell Specac P/N 5850 without windows was used to heat the sample up to 800 K.

Dielectric measurements in the high-frequency range were taken on two cylindrical samples cut from the bulk (of heights and diameters *h*_1_ = 4.0 mm, *d*_1_ = 0.95 mm and *h*_2_ = 7.0 mm, *d*_1_ = 0.8 mm) with the polar axis along the cylinder main axis. A computer controlled high-frequency dielectric spectrometer equipped with HP 4291B impedance analyser, a Novocontrol BDS 2100 coaxial sample cell and a Sigma System M18 chamber (temperature range 100–570 K) were used. Au electrodes were sputtered on the bases of the cylinders and the impedance of the samples was recorded on cooling at a temperature rate of 1 K/min. Results from experiments on both samples were merged.

Low-frequency dielectric measurements were performed with a Hewlett-Packard 4192 A impedance analyser. Gold electrodes were added onto the faces of a plate (4.5 × 5 × 0.8 mm^3^) with orientation (001) and also to the ends of one of the cylindrical samples (*h*_1_ = 4.0 mm, *d*_1_ = 0.95 mm). The samples were heated and cooled in the temperature range of 80–600 K and 80–450 K, respectively, at a temperature rate of 2 K/min under a measuring field of 5 V/cm.

### Data availability statement

All data generated or analysed during this study are included in this published article.
